# Poly[bis(2,2′-bipyridine-κ^2^ *N*,*N*′)heptadeca-μ-oxido-tetraoxidodicopper(II)divanadate(IV)hexavanadate(V)]

**DOI:** 10.1107/S1600536809052118

**Published:** 2009-12-09

**Authors:** Hai-hui Yu, Li Kong

**Affiliations:** a College of Chemical Engineering, North East Dianli University, Jilin 132012, People’s Republic of China; b Jilin Institute of Chemical Technology, Jilin 132012, People’s Republic of China

## Abstract

In the title complex, [Cu_2_V_8_O_21_(2,2′-bpy)_2_]_*n*_ (bpy = bipyridine, C_10_H_8_N_2_), the asymmetric unit contains four independent V atoms briged by 11 O atoms, one of which lies on an inversion center, and a [Cu(2,2′-bpy)]^2+^ unit. Three V atoms in the polyoxoanion exhibit distorted tetra­hedral coordination geometries while the fourth V atom adopts a trigonal-bipyramidal geometry. The Cu atom adopts a square-pyramidal geometry being coordinated by two nitro­gen donors of a 2,2′-bpy ligand, and three bridging O atoms which are linked with V atoms. The V_8_ polyoxoanion is connected to [Cu(2,2′-bpy)]^2+^ cations, resulting in a two-dimensional layer structure extending parallel to (010). C—H⋯O hydrogen bonding consolidates the structure.

## Related literature

For hybrid organic-inorganic vanadium oxides, see: Zapf *et al.* (1997 ▶); Liu *et al.* (2001 ▶, 2002 ▶); Yuan *et al.* (2002 ▶). For the organic substituents, see: Girginova *et al.* (2005 ▶); Paz & Klinowski (2003 ▶); Shi *et al.* (2005 ▶). 
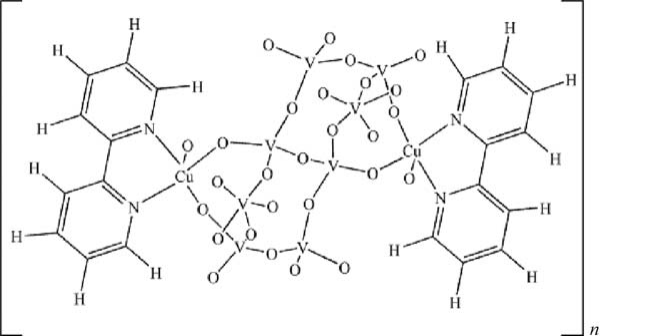


## Experimental

### 

#### Crystal data

[Cu_2_V_8_O_21_(C_10_H_8_N_2_)_2_]
*M* *_r_* = 591.48
Triclinic, 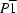

*a* = 8.0721 (16) Å
*b* = 9.764 (2) Å
*c* = 11.607 (2) Å
α = 85.58 (3)°
β = 72.79 (3)°
γ = 72.28 (3)°
*V* = 832.4 (3) Å^3^
*Z* = 2
Mo *K*α radiation
μ = 3.48 mm^−1^
*T* = 293 K
0.20 × 0.18 × 0.16 mm

#### Data collection

Rigaku R-AXIS RAPID diffractometer
Absorption correction: multi-scan (*ABSCOR*; Higashi, 1995 ▶) *T*_min_ = 0.504, *T*_max_ = 0.573
7138 measured reflections
3261 independent reflections
2916 reflections with *I* > 2σ(*I*)
*R*_int_ = 0.018

#### Refinement

*R*[*F* ^2^ > 2σ(*F* ^2^)] = 0.024
*wR*(*F* ^2^) = 0.056
*S* = 1.06
3261 reflections
282 parameters
H atoms treated by a mixture of independent and constrained refinement
Δρ_max_ = 0.39 e Å^−3^
Δρ_min_ = −0.37 e Å^−3^

### 

Data collection: *RAPID-AUTO* (Rigaku, 1998 ▶); cell refinement: *RAPID-AUTO*; data reduction: *RAPID-AUTO*; program(s) used to solve structure: *SHELXS97* (Sheldrick, 2008 ▶); program(s) used to refine structure: *SHELXL97* (Sheldrick, 2008 ▶); molecular graphics: *ORTEP-3* (Burnett & Johnson, 1996 ▶); software used to prepare material for publication: *SHELXTL97*.

## Supplementary Material

Crystal structure: contains datablocks I, New_Global_Publ_Block. DOI: 10.1107/S1600536809052118/pv2241sup1.cif

Structure factors: contains datablocks I. DOI: 10.1107/S1600536809052118/pv2241Isup2.hkl

Additional supplementary materials:  crystallographic information; 3D view; checkCIF report

## Figures and Tables

**Table 1:** Hydrogen-bond geometry (Å, °)

*D*—H⋯*A*	*D*—H	H⋯*A*	*D*⋯*A*	*D*—H⋯*A*
C1—H1⋯O4	0.94 (3)	2.53 (3)	3.068 (4)	117 (2)
C2—H2⋯O6^i^	0.91 (3)	2.57 (3)	3.132 (4)	121 (2)
C4—H4⋯O10^ii^	0.89 (3)	2.53 (3)	3.330 (4)	150 (3)
C7—H5⋯O9^ii^	0.91 (3)	2.59 (3)	3.306 (4)	136 (3)
C9—H7⋯O6^iii^	0.89 (3)	2.36 (3)	3.216 (4)	160 (3)
C10—H8⋯O3	0.96 (3)	2.36 (3)	2.937 (4)	118 (2)

Symmetry codes: (i) 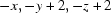; (ii) 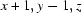; (iii) 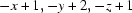.

**Fig. 1.**
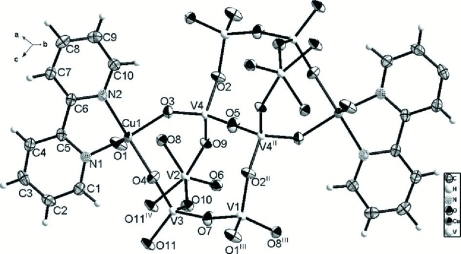
ORTEP drawing of the title compound with thermal ellipsoids at 50% probability. [symmetry codes: (i) 1 - x, 2 - y,1 - z; (ii) - x, 2 - y, 1 - z; (iii) -1 + x, y, z; (iv) - x, 2 - y, 2 - z.]

**Fig. 2.**
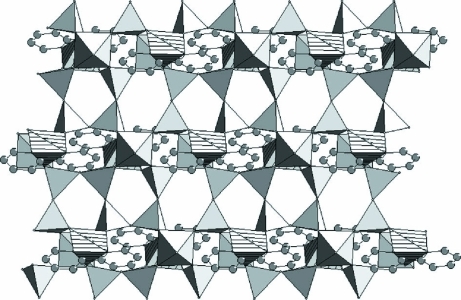
A polyhedral representation of two-dimensional layer-like structure of the title compound. All of the hydrogen atoms are omitted forclarity. [symmetry codes: (i) 1 - x, 2 - y, 1 - z; (ii) - x, 2 - y, 1 - z; (iii) -1 + x, y, z; (iv) - x, 2 - y, 2 - z; (v) 1 + x, y, z.]

## supplementary crystallographic information

### Comment

The introduction of hydrothermal technique and the use of organic groups which were performed not only as template agents, but also as ligands directly coordinated to the inorganic units, have led to the production of various organic-inorganic hybrid vanadium oxides with discrete clusters, one-dimensional, two-dimensional and three-dimensional structures (Zapf *et al.*, 1997; Liu *et al.*, 2001; Liu *et al.*, 2002; Yuan *et al.*,2002). Typically, the organic substituents have been presented as charge-compensating cations and structural filling agents. Examples of such compounds include the one-dimensional and the two-dimensional phases (Paz *et al.*, 2003; Girginova *et al.*, 2005, Shi *et al.*, 2005). Following interest in the hydrothermal approach to the synthesis of this family of hybrid compounds, we have synthesized a novel organic-inorganic hybrid vanadium oxide complexed with [Cu(2,2'-bpy)]^2+^ cation (bpy = bipyridine), [{Cu(2,2'-bpy)}_2_V_8_O_21_], (I). In this article, the crystal structure of the title compound is presented.

The crystals of the title compound (Fig. 1) consist of an unusual two-dimensional layer-like structure grafted with [Cu(2,2'-bpy)]^2+^ complex. The asymmetric unit of (I) contsians four crystallographically independent V atoms briged by 11 oxygen atoms, one of which (O5) lies on an inversion center and a [Cu(2,2'-bpy)]^2+^ complex. The atoms V1, V3 and V4 exhibit distorted tetrahedral coordination geometry coordinated with four bridging oxygen atoms. V1 and V4 share oxygen atoms with one {CuN_2_O_3_} square pyramid unit, one {VO_5_} square pyramid unit and two {VO_4_} tetrahedra, while V3 shares oxygen atoms with one {CuN_2_O_3_} square pyramid unit, two {VO_5_} square pyramids and one {VO_4_} tetrahedron. The atom V2 shows a trigonal bipyramidal coordination geometry with O9 and O11 atoms in axial and O6, O8 and O10 atoms at equatorial positions. With the exception of O6, the remaining four oxygen atoms are linked with two symmetry related V3 atoms and another two are linked with V1 and V4. The Cu atom adopts a square pyramidal geometry being coordinated by two nitrogen donors of a 2,2'-bpy ligand, and three bridging oxygen atoms which are linked with V1, V3, and V4, respectively. As shown in Fig. 2, two {VO_4_} tetrahedra shared a corner to give rise to a V_2_O_7_ moiety, which further produce a {V_8_O_21_}_n_ layer. Interestingly, each {CuN_2_O_3_} square pyramid attaches to three {VO_4_} tetrahedra of the vanadate layer *via* corner-sharing interaction. Therefore, the {[Cu(2,2'-bpy)]_2_V_8_O_21_}_n_ layer consists of 4-, 5- and 6- membered rings. The adjacent layers are stably packed together and exhibit an interesting three-dimensional supramolecular architecture.

### Experimental

The title compound was hydrothermally synthesized under autogenous pressure. A mixture of V_2_O_5_(0.66 g,3.6 mmol), As_2_O_3_(0.48 g,2.4 mmol), CuSO_4_.5H_2_O(0.75 g, 3 mmol), 2,2'-bipy (0.18 g, 1.2 mmol), 4,4'-bipy (0.24 g, 1.2 mmol) and 18 ml water was stirred for 120 min in air; it was adjusted to pH = 6.5 with 2*M* KOH, and was heated in a 25-ml stainless steel reactor with a Teflon-liner at 453 K for 3 days, and then cooled to room temperature. The resulting product consisting of brown block crystals was isolated by filtration, washed with distilled water, and dried at ambient temperature (51% yield based on V).

### Refinement

The H atoms were located from difference Fourier maps and were allowed to refine with isotropic displacement factors.

### Crystal data

**Table:**

[Cu_2_V_8_O_21_(C_10_H_8_N_2_)_2_]	*Z* = 2
*M**_r_* = 591.48	*F*(000) = 574
Triclinic, *P*1	*D*_x_ = 2.360 Mg m^−^^3^
Hall symbol: -P 1	Mo *K*α radiation, λ = 0.71073 Å
*a* = 8.0721 (16) Å	Cell parameters from 3536 reflections
*b* = 9.764 (2) Å	θ = 2.8–26.1°
*c* = 11.607 (2) Å	µ = 3.48 mm^−^^1^
α = 85.58 (3)°	*T* = 293 K
β = 72.79 (3)°	Block, brown
γ = 72.28 (3)°	0.20 × 0.18 × 0.16 mm
*V* = 832.4 (3) Å^3^	

### Data collection

**Table:**

Rigaku R-AXIS RAPID diffractometer	3261 independent reflections
Radiation source: fine-focus sealed tube	2916 reflections with *I* > 2σ(*I*)
graphite	*R*_int_ = 0.018
Detector resolution: 10 pixels mm^-1^	θ_max_ = 26.1°, θ_min_ = 2.2°
ω scans	*h* = −9→9
Absorption correction: multi-scan (*ABSCOR*; Higashi, 1995)	*k* = −12→12
*T*_min_ = 0.504, *T*_max_ = 0.573	*l* = −14→14
7138 measured reflections	

### Refinement

**Table:**

Refinement on *F*^2^	Primary atom site location: structure-invariant direct methods
Least-squares matrix: full	Secondary atom site location: difference Fourier map
*R*[*F*^2^ > 2σ(*F*^2^)] = 0.024	Hydrogen site location: inferred from neighbouring sites
*wR*(*F*^2^) = 0.056	H atoms treated by a mixture of independent and constrained refinement
*S* = 1.06	*w* = 1/[σ^2^(*F*_o_^2^) + (0.0235*P*)^2^ + 0.7292*P*] where *P* = (*F*_o_^2^ + 2*F*_c_^2^)/3
3261 reflections	(Δ/σ)_max_ = 0.001
282 parameters	Δρ_max_ = 0.39 e Å^−^^3^
0 restraints	Δρ_min_ = −0.37 e Å^−^^3^

### Special details

**Table:**

Geometry. All esds (except the esd in the dihedral angle between two l.s. planes) are estimated using the full covariance matrix. The cell esds are taken into account individually in the estimation of esds in distances, angles and torsion angles; correlations between esds in cell parameters are only used when they are defined by crystal symmetry. An approximate (isotropic) treatment of cell esds is used for estimating esds involving l.s. planes.
Refinement. Refinement of F^2^ against ALL reflections. The weighted R-factor wR and goodness of fit S are based on F^2^, conventional R-factors R are based on F, with F set to zero for negative F^2^. The threshold expression of F^2^ > 2sigma(F^2^) is used only for calculating R-factors(gt) etc. and is not relevant to the choice of reflections for refinement. R-factors based on F^2^ are statistically about twice as large as those based on F, and R- factors based on ALL data will be even larger.

### Fractional atomic coordinates and isotropic or equivalent isotropic displacement parameters (Å^2^)

**Table:**

	*x*	*y*	*z*	*U*_iso_*/*U*_eq_	
C1	0.2395 (4)	0.5437 (3)	0.9302 (3)	0.0289 (7)	
C2	0.2805 (5)	0.4323 (3)	1.0073 (3)	0.0320 (7)	
C3	0.4432 (5)	0.3260 (3)	0.9710 (3)	0.0317 (7)	
C4	0.5612 (4)	0.3335 (3)	0.8579 (3)	0.0274 (6)	
C5	0.5109 (4)	0.4469 (3)	0.7846 (2)	0.0198 (6)	
C6	0.6235 (4)	0.4644 (3)	0.6616 (2)	0.0201 (6)	
C7	0.7827 (4)	0.3634 (3)	0.6024 (3)	0.0284 (7)	
C8	0.8728 (4)	0.3894 (3)	0.4858 (3)	0.0322 (7)	
C9	0.8050 (4)	0.5152 (3)	0.4314 (3)	0.0306 (7)	
C10	0.6460 (4)	0.6144 (3)	0.4955 (3)	0.0265 (6)	
Cu1	0.31722 (4)	0.71169 (3)	0.70685 (3)	0.01922 (9)	
H1	0.128 (4)	0.617 (3)	0.952 (3)	0.029 (8)*	
H2	0.200 (4)	0.430 (3)	1.081 (3)	0.029 (8)*	
H3	0.472 (4)	0.254 (4)	1.021 (3)	0.033 (9)*	
H4	0.668 (4)	0.267 (3)	0.832 (3)	0.029 (8)*	
H5	0.823 (4)	0.284 (4)	0.644 (3)	0.036 (9)*	
H6	0.987 (4)	0.315 (3)	0.445 (3)	0.029 (8)*	
H7	0.865 (4)	0.532 (3)	0.356 (3)	0.027 (8)*	
H8	0.591 (4)	0.703 (3)	0.461 (3)	0.026 (8)*	
N1	0.3521 (3)	0.5516 (2)	0.8213 (2)	0.0210 (5)	
N2	0.5557 (3)	0.5887 (2)	0.6079 (2)	0.0205 (5)	
O1	0.4258 (3)	0.8537 (2)	0.79009 (18)	0.0333 (5)	
O2	0.3334 (3)	1.0721 (2)	0.41281 (17)	0.0276 (4)	
O3	0.3006 (3)	0.8439 (2)	0.57234 (18)	0.0270 (4)	
O4	0.0648 (3)	0.8000 (2)	0.79171 (18)	0.0296 (5)	
O5	0.0000	1.0000	0.5000	0.0330 (7)	
O6	0.0361 (3)	1.3383 (2)	0.81792 (17)	0.0274 (5)	
O7	−0.3076 (3)	0.9544 (2)	0.82587 (18)	0.0308 (5)	
O8	0.3910 (2)	1.1332 (2)	0.75539 (17)	0.0262 (4)	
O9	0.1379 (3)	1.1176 (2)	0.65337 (16)	0.0244 (4)	
O10	−0.0633 (3)	1.0792 (2)	0.87494 (17)	0.0247 (4)	
O11	−0.1608 (3)	0.8630 (2)	1.02173 (17)	0.0284 (5)	
V1	0.54061 (6)	0.96749 (5)	0.74290 (4)	0.01637 (11)	
V2	0.12848 (6)	1.16967 (5)	0.81859 (4)	0.01575 (10)	
V3	−0.11097 (6)	0.92167 (5)	0.87896 (4)	0.01417 (10)	
V4	0.19437 (6)	1.00633 (5)	0.53774 (4)	0.01430 (10)	

### Atomic displacement parameters (Å^2^)

**Table:**

	*U*^11^	*U*^22^	*U*^33^	*U*^12^	*U*^13^	*U*^23^
C1	0.0296 (16)	0.0257 (16)	0.0275 (16)	−0.0045 (14)	−0.0062 (13)	0.0000 (13)
C2	0.0417 (19)	0.0330 (17)	0.0197 (16)	−0.0140 (15)	−0.0046 (14)	0.0047 (13)
C3	0.0436 (19)	0.0250 (16)	0.0297 (17)	−0.0111 (14)	−0.0167 (15)	0.0108 (13)
C4	0.0263 (16)	0.0228 (15)	0.0316 (17)	−0.0029 (13)	−0.0113 (13)	0.0033 (12)
C5	0.0228 (14)	0.0174 (13)	0.0222 (14)	−0.0052 (11)	−0.0113 (11)	−0.0006 (11)
C6	0.0199 (14)	0.0171 (13)	0.0243 (14)	−0.0036 (11)	−0.0095 (11)	−0.0003 (11)
C7	0.0241 (15)	0.0252 (16)	0.0335 (17)	−0.0007 (13)	−0.0115 (13)	0.0008 (13)
C8	0.0220 (15)	0.0311 (17)	0.0360 (18)	−0.0008 (13)	−0.0030 (14)	−0.0057 (14)
C9	0.0281 (16)	0.0340 (17)	0.0266 (17)	−0.0104 (14)	−0.0017 (13)	−0.0011 (13)
C10	0.0281 (16)	0.0274 (16)	0.0238 (15)	−0.0085 (13)	−0.0079 (13)	0.0045 (12)
Cu1	0.01834 (17)	0.01617 (17)	0.02058 (18)	−0.00094 (13)	−0.00635 (13)	0.00129 (13)
N1	0.0232 (12)	0.0174 (11)	0.0210 (12)	−0.0046 (9)	−0.0061 (10)	0.0021 (9)
N2	0.0209 (12)	0.0172 (11)	0.0233 (12)	−0.0040 (9)	−0.0080 (10)	0.0016 (9)
O1	0.0460 (13)	0.0461 (13)	0.0204 (10)	−0.0322 (11)	−0.0096 (10)	0.0041 (9)
O2	0.0322 (11)	0.0334 (11)	0.0166 (10)	−0.0143 (9)	−0.0020 (8)	0.0021 (8)
O3	0.0280 (11)	0.0242 (10)	0.0260 (11)	−0.0007 (9)	−0.0119 (9)	0.0042 (8)
O4	0.0204 (10)	0.0293 (11)	0.0314 (12)	0.0006 (9)	−0.0038 (9)	−0.0022 (9)
O5	0.0244 (15)	0.0468 (19)	0.0342 (17)	−0.0099 (14)	−0.0179 (13)	−0.0021 (14)
O6	0.0340 (11)	0.0217 (10)	0.0213 (10)	−0.0047 (9)	−0.0038 (9)	0.0000 (8)
O7	0.0211 (10)	0.0457 (13)	0.0304 (12)	−0.0080 (9)	−0.0159 (9)	−0.0024 (10)
O8	0.0198 (10)	0.0335 (11)	0.0233 (10)	−0.0052 (9)	−0.0064 (8)	0.0014 (9)
O9	0.0282 (11)	0.0295 (11)	0.0163 (10)	−0.0069 (9)	−0.0084 (8)	−0.0033 (8)
O10	0.0246 (10)	0.0259 (10)	0.0267 (11)	−0.0124 (9)	−0.0075 (8)	0.0029 (8)
O11	0.0348 (12)	0.0411 (12)	0.0181 (10)	−0.0201 (10)	−0.0132 (9)	0.0078 (9)
V1	0.0148 (2)	0.0247 (2)	0.0129 (2)	−0.00865 (19)	−0.00619 (17)	0.00187 (17)
V2	0.0192 (2)	0.0173 (2)	0.0117 (2)	−0.00599 (18)	−0.00523 (17)	0.00065 (17)
V3	0.0121 (2)	0.0199 (2)	0.0120 (2)	−0.00499 (17)	−0.00582 (16)	0.00196 (17)
V4	0.0136 (2)	0.0184 (2)	0.0109 (2)	−0.00274 (17)	−0.00571 (17)	0.00037 (16)

### Geometric parameters (Å, °)

**Table:**

C1—N1	1.333 (4)	Cu1—N2	1.991 (2)
C1—C2	1.378 (4)	Cu1—O1	2.252 (2)
C1—H1	0.94 (3)	O1—V1	1.621 (2)
C2—C3	1.375 (5)	O2—V4	1.763 (2)
C2—H2	0.91 (3)	O2—V1^i^	1.797 (2)
C3—C4	1.387 (4)	O3—V4	1.6398 (19)
C3—H3	0.90 (3)	O4—V3	1.661 (2)
C4—C5	1.379 (4)	O5—V4^ii^	1.7684 (6)
C4—H4	0.89 (3)	O5—V4	1.7684 (6)
C5—N1	1.348 (3)	O6—V2	1.588 (2)
C5—C6	1.477 (4)	O7—V1^iii^	1.7405 (19)
C6—N2	1.357 (3)	O7—V3	1.8003 (19)
C6—C7	1.381 (4)	O8—V1	1.686 (2)
C7—C8	1.379 (4)	O8—V2	1.953 (2)
C7—H5	0.91 (3)	O9—V4	1.6586 (19)
C8—C9	1.372 (4)	O9—V2	1.9957 (19)
C8—H6	0.99 (3)	O10—V3	1.6894 (19)
C9—C10	1.389 (4)	O10—V2	1.936 (2)
C9—H7	0.89 (3)	O11—V3	1.6840 (19)
C10—N2	1.340 (4)	O11—V2^iv^	1.9363 (19)
C10—H8	0.96 (3)	V1—O7^v^	1.7405 (19)
Cu1—O4	1.934 (2)	V1—O2^i^	1.797 (2)
Cu1—O3	1.957 (2)	V2—O11^iv^	1.9363 (19)
Cu1—N1	1.980 (2)		
			
N1—C1—C2	122.2 (3)	C5—N1—Cu1	114.58 (18)
N1—C1—H1	116.7 (19)	C10—N2—C6	119.3 (2)
C2—C1—H1	121.0 (19)	C10—N2—Cu1	126.54 (19)
C3—C2—C1	118.9 (3)	C6—N2—Cu1	114.11 (18)
C3—C2—H2	121 (2)	V1—O1—Cu1	136.75 (11)
C1—C2—H2	120 (2)	V4—O2—V1^i^	142.02 (12)
C2—C3—C4	119.2 (3)	V4—O3—Cu1	143.18 (12)
C2—C3—H3	119 (2)	V3—O4—Cu1	156.12 (13)
C4—C3—H3	121 (2)	V4^ii^—O5—V4	180.0
C5—C4—C3	118.9 (3)	V1^iii^—O7—V3	166.30 (13)
C5—C4—H4	120 (2)	V1—O8—V2	123.33 (11)
C3—C4—H4	121 (2)	V4—O9—V2	154.58 (12)
N1—C5—C4	121.5 (3)	V3—O10—V2	143.70 (12)
N1—C5—C6	114.8 (2)	V3—O11—V2^iv^	153.85 (12)
C4—C5—C6	123.7 (3)	O1—V1—O8	107.63 (11)
N2—C6—C7	121.4 (3)	O1—V1—O7^v^	110.71 (11)
N2—C6—C5	114.4 (2)	O8—V1—O7^v^	111.38 (10)
C7—C6—C5	124.2 (3)	O1—V1—O2^i^	108.79 (10)
C8—C7—C6	119.0 (3)	O8—V1—O2^i^	109.54 (10)
C8—C7—H5	124 (2)	O7^v^—V1—O2^i^	108.75 (10)
C6—C7—H5	117 (2)	O6—V2—O11^iv^	103.25 (10)
C9—C8—C7	119.8 (3)	O6—V2—O10	107.76 (10)
C9—C8—H6	122.7 (18)	O11^iv^—V2—O10	86.50 (9)
C7—C8—H6	117.5 (18)	O6—V2—O8	107.90 (10)
C8—C9—C10	119.0 (3)	O11^iv^—V2—O8	87.79 (9)
C8—C9—H7	119 (2)	O10—V2—O8	144.26 (9)
C10—C9—H7	122 (2)	O6—V2—O9	99.91 (10)
N2—C10—C9	121.5 (3)	O11^iv^—V2—O9	156.82 (9)
N2—C10—H8	116.0 (18)	O10—V2—O9	85.62 (9)
C9—C10—H8	122.4 (18)	O8—V2—O9	85.96 (9)
O4—Cu1—O3	91.37 (9)	O4—V3—O11	110.09 (11)
O4—Cu1—N1	94.77 (10)	O4—V3—O10	110.01 (10)
O3—Cu1—N1	170.15 (9)	O11—V3—O10	110.08 (10)
O4—Cu1—N2	167.12 (9)	O4—V3—O7	110.49 (10)
O3—Cu1—N2	90.36 (9)	O11—V3—O7	108.55 (10)
N1—Cu1—N2	81.96 (10)	O10—V3—O7	107.58 (10)
O4—Cu1—O1	95.62 (9)	O3—V4—O9	109.67 (10)
O3—Cu1—O1	90.80 (8)	O3—V4—O2	111.30 (10)
N1—Cu1—O1	96.24 (9)	O9—V4—O2	107.76 (10)
N2—Cu1—O1	97.12 (9)	O3—V4—O5	108.67 (8)
C1—N1—C5	119.1 (2)	O9—V4—O5	111.12 (7)
C1—N1—Cu1	126.2 (2)	O2—V4—O5	108.34 (7)

Symmetry codes: (i) −*x*+1, −*y*+2, −*z*+1; (ii) −*x*, −*y*+2, −*z*+1; (iii) *x*−1, *y*, *z*; (iv) −*x*, −*y*+2, −*z*+2; (v) *x*+1, *y*, *z*.

### Hydrogen-bond geometry (Å, °)

**Table:**

*D*—H···*A*	*D*—H	H···*A*	*D*···*A*	*D*—H···*A*
C1—H1···O4	0.94 (3)	2.53 (3)	3.068 (4)	117 (2)
C2—H2···O6^iv^	0.91 (3)	2.57 (3)	3.132 (4)	121 (2)
C4—H4···O10^vi^	0.89 (3)	2.53 (3)	3.330 (4)	150 (3)
C7—H5···O9^vi^	0.91 (3)	2.59 (3)	3.306 (4)	136 (3)
C9—H7···O6^i^	0.89 (3)	2.36 (3)	3.216 (4)	160 (3)
C10—H8···O3	0.96 (3)	2.36 (3)	2.937 (4)	118 (2)

Symmetry codes: (iv) −*x*, −*y*+2, −*z*+2; (vi) *x*+1, *y*−1, *z*; (i) −*x*+1, −*y*+2, −*z*+1.
